# “Tumor Immunology Meets Oncology” (TIMO), 22 May–24 May 2025 in Brandenburg an der Havel, Germany

**DOI:** 10.1007/s00262-026-04468-y

**Published:** 2026-06-23

**Authors:** Barbara Seliger

**Affiliations:** 1 Brandenburg Medical School (Theodor Fontane) and Faculty of Health Sciences Brandenburg, Institute of Translational Immunology, Gertrud-Piter-Platz7, 14770 Brandenburg an Der Havel, Germany; 2 Institute of Pathology, Martin Luther University of Halle-Wittenberg, Section Immunopathology, Magdeburger Straße 02, 06112 Halle (Saale), Germany; 3 Fraunhofer Institute for Cell Therapy and Immunology (IZI), Leipzig, Germany

**Keywords:** Immune evasion, Antitumoral immune response, Biomarkers, Immunotherapy, TIMO 2025, Tumor microenvironment

## Abstract

The “Tumor Immunology Meets Oncology” (TIMO XIX) meeting 2025 reports on the rapidly growing field of basic, but in particular translational tumor immunology thereby providing deeper insights into the heterogeneity of tumors, their microenvironment and clinical relevance. This represents the basis for the development of novel immunotherapeutic strategies and combinatorial therapies.

## Introduction

The annual meeting “Tumor Immunology Meets Oncology” (TIMO XIX) 2025 took place at the City Hall of Brandenburg an der Havel, Germany. The main symposium was coupled with a TIMO workshop allowing junior scientist and clinicians to present their own data in short talks or posters and with a workshop on “Cancer and Inflammation” hosted by the Brandenburg Medical School in association with the Faculty of Health Sciences in Brandenburg an der Havel, Germany. As in the previous TIMO meetings, there were a large number of national, but also international scientists and clinicians, but also junior researchers participated and discussed the increasing knowledge of tumor immunology as well as of immunotherapies. Since it has become obvious that the tumor development is not only associated cellular and/or genetic events, the meeting focuses on the interaction between host and tumor cells by in depth characterization of the complex tumor ecosystem using different “omics”-based approaches. This comprises next to tumor cells, the tumor microenvironment (TME) characterized by different immune cell subpopulations, blood vessels, extracellular matrix (ECM), non-immune cells, like, e.g., cancer-associated fibroblasts (CAFs) and blood vessels, as well as different soluble components such as cytokines and metabolites. In addition, analysis of blood samples from tumor patients prior, during and after therapy resulted in the identification of novel targets and predictive and prognostic biomarkers, which increases the knowledge of tumor initiation and progression as well as therapy resistance. The development of different bioinformatics tools provided further insights into the biology of cancer, which also allow the integration of high dimensional data into a holistic network of the malignancy. These different strategies present potential opportunities for the design and development of novel therapies.

In total, the symposium had 24 speakers from 9 countries including the United States, Great Britain, Israel, Portugal, Denmark, Sweden, Germany, Italy and Austria. It was divided into different sessions with specific topics ranging from the characterization of immune escape mechanisms and their reversion, the modulation of the TME, identification of novel immunotherapeutic targets as well as the development and improvement of immunotherapies alone or in combination with other treatments.

### Clinical and translational highlight

The clinical and translational highlight of the TIMO meeting was presented by the keynote speaker Sergio Quezada from the University College London, London (Great Britain), who talked about targeting regulatory T cells (Tregs) in solid tumors. The antitumoral activity of the monoclonal antibody (mAb) directed against the cytotoxic T lymphocyte associated protein 4 (CTLA-4) is mediated by the engagement of the Fc receptor (FcR) leading to the depletion of Tregs, which has an impact on the innate immune responses due to the activation of the type I interferon (IFN) signaling pathway in the myeloid compartment. Since CD25 is primarily expressed on Tregs, the IL-2 receptor (IL-2R) CD25 is a better target for Treg depletion than CTLA-4. Indeed, the anti-CD25 mAb has been shown to be more effective against tumors than the anti-CTLA-4 mAb. Based on these data, a non-interleukin blocking anti-human CD25 mAb (CD25^NIB^) with the capacity to preferentially deplete dose-dependent Tregs in peripheral mononuclear cells (PBMCs) and human tumor samples was developed. This Ab induced an inflamed tumor type by promoting CD8^+^ T cell expansion, upregulating programmed death receptor ligand 1 (PD-L1) as well as relocating CD8^+^ and FoxP3^+^ T cells to the inner part of the tumor. Since a Treg signature has been shown to be associated with a worse patients’ survival, the effect of Treg depletion on glioblastoma progression and therapy response was determined. Indeed, in an experimental glioblastoma model, the anti-CD25^NIB^ mAb promotes effective Treg depletion and the clonal expansion of effector subpopulations. This was due to the induction of a transcriptional program across multiple subsets of tumor infiltrating CD8^+^ T cells, which enhanced the IL-2- and CD8-dependent potent antitumoral activity and modulated the IFN-γ-dependent myeloid compartment. These important experimental data were then translated into the human system using glioblastoma tumor explants. Furthermore, complete responses were obtained upon treatment of glioblastoma with a combination of anti-CD25^NIB^ and anti-EGFR VIII mAbs. In sum, these data provide insights on the impact of Treg depletion on the TME via anti-CD25^NIB^ and the growing potential of this approach for combination therapies.

### Immune escape mechanisms and their interventions

Barbara Seliger talked about immune escape as one major hallmark of cancer and as cause of the limited response to immunotherapies. Indeed, tumors develop different strategies to evade immune surveillance. These include abnormalities of the human leukocyte antigens (HLA) class I present in different tumor (sub)types, but to a distinct extent as representatively shown for breast cancer (BC). High levels and/or amplification of HER-2 expression in BC were inversely correlated with HLA class I surface expression. Regional and ethnical differences of HLA-I expression in samples of Sub-Saharan Africa were found, which were associated with an altered immune cell infiltration, pathogen infection and patients’ survival. The underlying mechanisms of HLA class I alterations range from structural alterations to deregulation at the transcriptional, epigenetic posttranscriptional as well as translational level. The team of B. Seliger has identified a number of microRNAs (miRNAs) affecting the expression of components of the HLA class I antigen processing machinery in different tumor types, such as, e.g., the transporter associated with antigen processing 1 and tapasin. Interestingly, miRNAs not only inhibit target genes but could also enhance their expression as demonstrated for miR-155 leading to an upregulation of tapasin expression. This was accompanied by a reduced NK cell response, increased HLA class I surface expression in tumor cells and an increased CD8^+^ T cell infiltration in tumors. Next to alterations in the tumor, changes in the TME were linked with the patients’ prognosis and survival. A high frequency of tumor infiltrating lymphocytes (TILs) directly correlated with a better patients’ survival. Furthermore, an altered immunogenicity of BC samples in patients infected with the human immunodeficiency virus infection was found, which was characterized by a distinct immune cell composition, IFN signaling, HLA class I expression and an overexpression of the immune checkpoint (ICP) CD276. Since only a limited number of patients does respond to immune checkpoint inhibitor (ICPi) therapy, an increased understanding of the modulators and cellular drivers of immune escape is urgently required. Indeed, downregulation or loss of components of the HLA-I APM or IFN signal transduction cascade were associated with a reduced response to ICPis and adoptive T cell therapy (ACT). In depth analysis of peripheral blood prior and during treatment of triple negative breast cancer (TNBC) with an anti-PD-L1 mAb in combination with different chemotherapeutics identified biomarkers, which predict therapy response at the recruitment, such as CD4^+^ T cells, and during therapy, like gamma delta T cells.

Nana Talvard-Balland from Robert Zeiser’s group of the University Hospital in Freiburg, Germany, provided insights into the role of immune escape in acute leukemia associated with genetic alterations of p53 as well as fms-like tyrosine kinase 3 (FLT3). The downregulation p53 expression in acute myeloid leukemia (AML) due to the overexpression of the negative regulator MDM2 was linked to low expression levels of HLA class II antigens and the class II major histocompatibility complex transactivator (CIITA). Interestingly, the MDM2 inhibitors RG7112 and HDM201 increase the expression of CIITA thereby enhancing the graft versus leukemia (GvL) effects. In addition, the link between FLT3, galectin 9 and the carcinoembryonic antigen cell adhesion molecule 1 was investigated. In particular, the FLT3-ITD mutational status and the expression levels of T cell immunoglobulin and mucin domain-3 (TIM-3) or its ligands are functionally connected in leukemia. Anti-TIM-3 Ab treatment of allo-hematopoietic stem cell transplantations (HSCTs) enhances GvL effects, which depends on oncogenic mutations. Treatment with an anti-TIM-3 Ab reduces T cell exhaustion both in vitro as well as in vivo*,* increases the glycolysis of T cells and enhances the numbers of macrophages, dendritic cells (DC) as well as neutrophils. Deletion of TIM-3 in CD8^+^ T cells results in their activation, accompanied by an altered proliferation and cytotoxicity, improved GvL effects, but no graft versus host disease (GvHD) development and expansion of precursor memory CD8^+^ T cells. Since the oncogene-induced TIM-3 ligand expression dictates the susceptibility to anti-TIM-3 therapy in mice, an inhibitor of TIM-3 might be used for the treatment of AML patients after allogenic stem cell transplantation, which is currently tested in a clinical trial.

Luis Zapata-Ortiz from the Institute of Cancer Research in London focused his talk on the development of an immunogenomic-based matrix to predict responses to immune checkpoint inhibitor therapies. Using bioinformatics analysis, he studied a large cohort of primary tumors from The Cancer Genome Atlas concerning non-synonymous and synonymous mutations. He identified the landscape of driver genes in tumors that escaped the immune systems in comparison to those that were recognized by the immune system. Despite a number of novel driver genes were identified in immune escape tumors using this approach, the number is significantly lower than that of non-escape tumors. Thus, there is an urgent need for a better patient stratification undergoing specific therapies, which also allow the development of novel therapies. Furthermore, the genomic intra-tumoral heterogeneity was mapped in locally advanced prostate cancers. The team identified the genetic and morphological diversity were independent predictors of disease progression. Copy number changes associated with tumor grade were identified as well as loss of chromosome 6p was correlated with a reduced immune cell infiltration. The combination of genomics with artificial intelligence guided histopathology might lead to the identification of clinical biomarkers of evolution. These results were also extended to immune selection, which determines tumor antigenicity and influences the response to ICPis. Interestingly, immune escape metastases experienced the best response to immunotherapy, while immune-edited patients did not benefit suggesting a pre-existing resistance mechanism. The presence of a genetic immune escape, a less cytotoxic T cell phenotype and a lack of clonal T cell expansion were linked to poor treatment response. Furthermore, the widespread transcriptional and environmental changes through treatment with limited clonal replacement suggested a phenotypic plasticity.

Lorenzo Galluzzi from the Fox Chase Cancer Center, Philadelphia, USA, reported about the effects of radiation therapy on antitumor immune responses, with a focus on the critical role of type I interferon (IFN). Type I IFN secretion in irradiated cells is elicited by mitochondrial outer membrane permeabilization (MOMP), enabling exposure of mitochondrial DNA to the cytosol and activation of cGAS signaling. These data suggest that cancer cells harness a mitochondrial ICP to suppress anticancer immunity, which could be therapeutically used to enhance the immunogenicity of radiotherapy. It has indeed been demonstrated that the selective BCL2 inhibitor venetoclax, which is currently approved for the treatment of some haematological cancer, can exacerbate radiotherapy-driven MOMP, a therapeutically relevant activity that will be investigated in clinical trial. Of note, autophagy also limits the immunological consequences of MOMP, inspiring the design of novel strategies to enhance the immunogenicity of radiotherapy. These results were proven by using autophagy-deficient cells, resulting not only in superior radiotherapy-driven cytotoxicity, but also in superior type I IFN secretion. Furthermore, a signature of autophagy had a negative prognostic value for the BC patients, which inversely correlated to type I IFN signaling and effector immunity.

The talk of Lorenzo Moretta from the Children’s Hospital Bambino Gesù in Roma, Italy, focused on natural killer (NK) cells for the therapy of high-risk leukemia, since human NK cell receptors and their cellular ligands are fundamental in tumor recognition and killing. Therefore, the role of NK cells in haplo-identical hematopoietic stem cell transplantation (haplo-HSCT), a therapeutic approach implemented for the therapy of high-risk pediatric leukemia, was analyzed. Of note, in an allogenic setting, a fraction of “donor A” NK cells may express receptors that are not engaged by the HLA-I alleles of “donor B.” In haplo-HSCT, donor NK cells are “alloreactive” for leukemia cells as demonstrated by leukemia-free survival of both acute lymphatic leukemia and AML patients. Importantly, many different mechanisms of inhibition of NK cells exist, which cause NK cell dysfunction. These include the expression of programmed death receptor 1 (PD-1) and IL-1R8 as well as the presence of polymorphonuclear myeloid-derived suppressor cells (PMN-MDSCs) that are present not only in the TME, but also in PBMCs. PD-1-expressing NK cells display an impaired antitumor activity. On the other hand, silencing of IL-1R8 improves the antitumor activity of human NK cells. Furthermore, PMN-MDSC have been shown to be highly enriched in PBMCs of neuroblastoma patients. These patients generally respond to GD2- specific CAR T cells, but not in the presence of high numbers of PMN-MDSCs in PBMCs. Remarkably, the number of NK cells inversely correlated with those of PMN-MDSCs in PBMCs suggesting an inhibitory effect of PMN-MDSC generation and circulation of NK cells. In addition, PMN-MDSCs impaired the cytotoxicity of NK cells might be capability in vitro. Thus, PMN-MDSC could represent an important biomarker suggesting an inhibitory effect on natural defenses and on adoptively transferred CAR-T cells. Accordingly, these cells may represent a suitable target to improve cell-based immunotherapies and the clinical outcome of tumor patients.

The role for NK cell reactivity in the TME of the hostile microenvironment of colorectal cancer (CRC) was investigated by Adelheid Cerwenka from the Mannheim Institute for Innate Immunoscience, Mannheim, Germany. This disease has limited treatment options and only mismatch repair deficient CRC could be effectively treated by T cell-based immunotherapies. Due to the high inter-patients’ variability and the lack of tools to study immune cell-mediated anti-CRC responses, a CRC patient-derived organoid (PDO) platform was developed. CRC PDOs resistant to NK cell-mediated killing display hypoxia and transforming growth factor (TGF)-β signatures, while inhibition of the TGF-β receptor 1 improves the elimination of CRC PDO by NK cells. Furthermore, PDO have a hypoxic TME, which inhibits human NK cell effector responses as determined by cytokine release direct antitumor response as well as proliferation. Deletion of the hypoxia-inducible factor (HIF)1A improves NK cell-mediated PDO killing, which was also confirmed in a HIF1A knockout mouse. In sum, CRC mimic a hypoxic and TGF-β-rich microenvironment suggesting that HIF1A as well as TGF-β represent general checkpoints for human NK cell activity and could be targeted to enhance NK cell reactivity. Furthermore, NK cells from PDO cultures align with NK cell features of CRC patients’ tissues. NK cell-susceptible PDOs induce inflammatory transcriptional programs in NK cells, NK cell activation as well as drive the diversification of NK cell activation states, which coincidence with the tissue-like phenotype. An altered NK cell ligand expression was found on PDOs resistant or susceptible to NK cell-mediated killing, in particular concerning MICA/B and HLA class I. Thus, there exists a link between patient-specific susceptibilities of individual PDOs and NK cell-mediated killing. The inflammation tissue-like signature NK cells PDO co-align with scRNAseq data of NK cells from CRC tissues. Therefore, this platform might serve as tool for preclinical testing of novel immunotherapeutic treatment strategies and allogenic NK cell products.

### Tumor microenvironment (TME) and metabolism

Shuji Ogino from the Harvard Medical School and Brigham and Women’s Hospital in Boston, USA, provided an integrative view of the role of the TME, microbiota and immunity in early onset cancer CRC with the major goal to increase the knowledge about cancer and to modify lifestyle in order to prevent cancer and improve therapy. The integration of epidemiology, pathology, microbiology, immunology and data science provides the framework in population approach based on the availability of long-term exposure data and long follow-up. In the Nurse’s Health Study (since 1976), NHS2, and Health Professionals Follow-up Study, more than 3500 colorectal adenoma–carcinoma tumors were collected across the USA and analyzed by genomics, epigenetics, microbiota, in situ single cell multispectral immunofluorescence analysis, artificial intelligence (AI) and spatial biology. Smoking which exerts an immune suppressive effect on the microenvironment characterized by fewer T cells and macrophages has been associated with the CRC incidence and high tumor mutational burden (TMB). Smoking appears to impair immune response, thereby promoting a high TMB. Concerning bacterial infections, microsatellite instability (MSI)-high tumors are associated with the abundance of *Fusobacterium nucleatum* and *Bacteroides fragilis*, possibly due to their immunosuppressive effects. CRC have developed different immune evasion strategies, such as immunosuppressive bacterial presence, impaired expression of HLA-I APM components, deregulation of ICP molecules and signal transduction pathways. Environmental risk factor exposures alter the TME and provide selective advantages for certain mutant cells and clones. In early onset CRC, high immune suppression with low numbers of TILs and lack of dominant T cell clones have been shown with an increase in “immune cold” young non-MSI CRC. In these tumors, the immune responses can be modulated and enhanced. In sum, molecular pathological epidemiology studies give insights in the immunopathology of tumors and environmental features including aspirin, diet, smoking, vitamin D, obesity and exercise, which can be linked to cancer with specific alterations of the tumor immune microenvironment. These insights give clues to how we can prevent and treat cancers.

Ravid Straussman from the Weizmann Institute in Rehovot, Israel, linked the microbiome composition of tumors with the response to immunotherapy. This is based on the fact that ICPi resistance is very common in different tumor types, including melanoma. Different underlying mechanisms associated with immunotherapy resistance include MHC class I alterations, immune suppressive cells, nutrients, signaling pathway mutations, chromatin remodeling as well as changes in microbiota. For example, the metabolism of tryptophan to kynurenine (Kyn) has been shown to reduce ICPi response due to activation of 2′5’ indoleamine (IDO), but inhibition of IDO in combination with an ICPi has no effects. The tumor microbiome is composed of tumor type-specific intracellular bacteria, which is further promoted by the identification of different microbial-derived, HLA-bound peptides in melanoma. The tumor microbiome affects the response to immunotherapy in melanoma, but also in CRC, lung cancer, TNBC as well as squamous cell carcinoma. A high microbial richness determined by RNA sequencing (RNAseq) and single cell RNA sequencing (scRNAseq) analysis is associated with a suppressed intra-tumoral immune landscape and directly correlates with a response to ICPi. In particular, multiple bacterial genes in the Kyn pathway are enriched in non-responders. The tryptophan metabolism by bacteria can affect tumor immunity suggesting that tryptophan degradation by bacteria may reduce a response to ICPi. Interestingly, inhibition of IDO-1 only partially resulted in a Kyn reduction in serum and tumor samples lesions with low CD8^+^ T cells are enriched by tryptophan degrading bacteria. A similar association was found for M1 macrophages. Since the currently available IDO inhibitors do not block the Kyn pathway, a triple inhibition of IDO/TDO/KynA has been suggested to improve the response to ICPi. Different KynA enzymes are found in bacteria suggesting novel mechanisms of bacterial-mediated resistance to ICPi therapy including changes of the tumor microbiome.

The role of bacterial infections in tumor progression and therapy response was also underlined by Ofer Mandelboim from the Hadassah University in Jerusalem, Israel. There exists accumulating evidence that *Fusobacterium nucleatum* is involved in the progression and metastasis formation of a number of tumor types and has been shown also to induce chemotherapy resistance. *Fusobacterium nucleatum* colonize extraoral tumor entities such as CRC and breast cancer thereby modulating the TME and inducing a proinflammatory profile by binding Siglet-7 via lipopolysaccharides. Furthermore, it could bind to Siglet-7 on NK cells, thereby inhibiting the NK cell-mediated elimination of tumor cells, which is dependent on the arginine residue R124 in Siglet-7. These data extend the role of *Fusobacterium nucleatum* concerning the immune inhibitory activities with T cell immunoglobulin and immunoreceptor tyrosine-based inhibitory motif (TIGIT) and carcinoembryonic antigen related adhesion molecule 1 (CEACAM 1) and yield the rational for specific treatment of *Fusobacterium nucleatum* bearing tumors.

### Biomarker discovery

Markus Maeurer from the Champalimaud Foundation in Lisbon, Portugal, focused his talk on T cell diversity in particular in the TME. Beyond the T cell receptor (TCR) repertoire diversity, TCR “network” analysis measures the number of closely related (single amino acid difference) TCR clonotypes. This reflects a per-determined convergent selection, which is naturally found in blood from healthy individuals defying generative null expectations. This has neither been further investigated in high content analysis of the TME nor in combination with specific MHC peptide recognition associated with functional Th1/2/17 phenotypes. A loss of complex TCR network connectivity was found in tissue from patients with gastrointestinal cancer, while it was increased in tissues obtained from autoimmune diseases. This raised the question whether immune exhaustion, tolerance or immune evasion may play a role in reshaping these TCR networks. The expansion of TILs can restore TCR networks, whose nodes are believed to share recognition potential (convergence). By using bioinformatic tools in an HLA matched system, TCR nodes were frequently identified, where antigen specificity is redundant from independent MHC peptide TCR matching.

Using MiXCR outputs, we build length-matched Hamming-1 graphs and tcrdist meta-clonotypes, benchmarked against OLGA/IGoR generative nulls to confirm the non-stochastic nature of TCR networks and calculated condition-specific enrichment with ALICE/TCRNET statistics to determine condition-specific enrichment across blood/tumor/TIL. TCR networks in tumor tissues, e.g., pancreatic ductal adenocarcinoma (PDAC), typically show reduced connectivity that is restored in TIL expansion. These metrics together with TCR/peptide-MHC matching may serve as compact, reproducible biomarkers of tumor-specific immunity and serve as surrogate markers for enriched, smart TIL cell products for patients with solid cancer.

In order to predict responses to immunotherapies, biomarkers are urgently needed. Sumit Subudhi from the MD Anderson Cancer Center in Houston, Texas (USA) characterized the heterogeneity of metastatic prostate cancer (PCa) and identified candidate clinical and immune biomarkers, which allowed to predict response to ICPis. Since the anti-CTLA-4 mAb ipilimumab had only a durable effect in some patients, this subset of PCa patients has to be identified. Analysis of the tumor infiltrating immune cells in pre-treatment samples demonstrated an association of a T cell, mature B cell, as well as tertiary lymphoid structure signatures with durable clinical responses. In addition, in on-treatment tissues, ipilimumab induced PD-L1 expression within the prostate TME. Based on these data, the combination of nivolumab plus ipilimumab was evaluated in patients with metastatic PCa (NCT02985957 [CheckMate 650] and NCT03651271 [AMADEUS]). The combination of nivolumab plus ipilimumab yielded a higher rate of clinical responses in patients with measurable soft tissue disease as opposed to those with predominant bone disease. In fact, the response rate in the predominant bone disease was similar to what has been observed with monotherapy. Assessment of on-treatment tissue biomarkers demonstrated increased frequency of intra-tumoral CD8^+^ T cells. Thus, the combination of nivolumab plus ipilimumab improved clinical responses in a subset of metastatic PCa patients. However, increased ipilimumab concentrations were associated with higher rates of treatment-related toxicities. Disease control was associated with lower tumor burden and pre-existing immune activation in the TME, while adaptive resistance was linked to an upregulation of TREM2^+^ myeloid cells. The use of bispecific PSMAxCD28 T cell bispecific engagers in combination with an anti-PD-1-mAb did overcome the immune suppressive TME of PCa with an enhanced antitumor activity. Since PSMAxCD28 dosing influences T cell activation and cytotoxicity, it is necessary to derisk immune oncology drug development and lower costs by improving patient selection and selecting the best drug dose based on safety, biological changes and efficacy. This led to the development of a trial (NCT06826768) implementing increasing dose levels PSMAxCD28 in combination with an anti-PD-1 mAb and monitoring of the safety, immune activity, and efficacy over time.

Hubert Hackl from the Institute of Bioinformatics Biocenter of the Medical University of Innsbruck, Austria, extended the identification of biomarkers by talking about determinants of response to immunotherapies in ovarian cancer (OC). Over decades, standard therapies were used for treatment of OC, while targeted therapies with polyadenosine-diphosphate-ribose polymerase (PARP) as well as angiogenesis inhibitors have been recently successfully implemented. In contrast, the objective response of OC patients to immunotherapy is approx. only 10%, but combinations of PARP inhibitors with ICPi increased response. BRCAness tumors exhibit an activation of immune-regulated pathways in particular of the IFN-α response and NFκB pathway, IRF3 targets as well as STING. The cGAS-STING pathway was activated in BRCAness OC cells and a combination of cGAS-STING with PD-1 inhibition resulted in an upregulation of genes involved in the IFN-α, JAK STAT, NFκB, as well as TNFα signaling pathways suggesting an activation of the innate immune system. Furthermore, patients with BRACness OC and a favorable balance between activated and suppressive TME had a better response to a combination therapy of PARP inhibitors with anti-PD-1 mAb, in which tumor-associated macrophages (TAMs) play a central role. Analysis of gene signatures predicted that targeted therapy may overcome T cell exclusion and induce favorable changes in the tumor microenvironment, leading to improved responses to immunotherapy. This has led to the development of additional predictive models for immunotherapy outcomes, employing network and systems biology approaches, blood-based strategies and meta-analyses, which are expected to be further expanded with AI-based models in future.

In addition, Simon Knott from the CEDARS-SINAI Hospital in Los Angeles (USA) presented in depth bioinformatics analyses of tissue samples from a study combining radiation with pembrolizumab in TNBC. This study is based on a recent trial of neoadjuvant chemotherapy with pembrolizumab, which increased the pathological complete response as well as the event-free survival in TBNC patients. Spatially defined cellular communities or districts in tumors of responders and non-responders were identified by S. Knott using RNAseq data, which allowed to correlate phenotypic measurements from different modalities. T cell expansion was correlated with an antigen-presenting macrophage niche and found in different tumor areas, which point to a heterogeneous responder group. After radiotherapy, some tumors gained antigen-presenting macrophages, which was accompanied with an effector expansion. Three distinct immunological response trajectories to pembrolizumab with radiation therapy were identified in TBNC. These data were extended to another study combining radiation therapy pembrolizumab in high risk BRCA-positive BC. Responsive tumors were enriched for fibroblasts and showed plasma B cell expansion prior to therapy, while non-responsive BC had a low immune activity and were in particular enriched for adipocytes. Furthermore, non-responder TNBC patients had no alterations in the immune cell activity after therapy.

### Novel (immuno)therapeutic strategies

A major topic of TIMO was the development of novel therapeutic targets and treatment strategies. Epigenetic changes, such as aberrant methylation, have been shown to be involved in cancer development and progression as well as in impaired immunogenicity and immune recognition of cancer cells. Therefore, targeting of the epigenome by, e.g., DNA hypomethylating agents, like azacitidine and decitabine has been recently implemented. Michele Maio from the University of Siena, Italy, demonstrated that epigenetic remodeling of cancer cells by these agents results in an upregulation and induction of different immune-relevant molecules including HLA class I antigens, costimulatory molecules as well as tumor-associated antigens. These phenotypic changes lead to an improved recognition by tumor antigen-restricted CD8^+^ CTL and to an induction of humoral immune responses in vivo. So far, these epigenetic drugs have been successfully used for the treatment of various diseases including myeloproliferative syndrome, AML, but also melanoma. Furthermore, the substances have been recently combined with different cancer immunotherapies to enhance T cell responses. In depth analyses of the transcriptional programs induced in melanoma cells by different epigenetic drugs demonstrated an upregulation of immune-related genes, which were predictive for the response to immunotherapy of this disease. Therefore, epigenetic drugs represent a promising strategy to increase the efficacy of cancer immunotherapies and novel epigenetic-based immunotherapeutic approaches for the treatment of solid tumors, including glioblastoma, should be developed.

The importance of exercise to enhance the activity of the immune system was reported by Per Thor Straten from the Center for Cancer Immunotherapy of the University of Copenhagen, Denmark. He showed that exercise improves immune responses by increasing common lymphoid progenitors, delay or reverse immune senescence and increase the removal senescent tissue cells. This enhances the quality of live, lowers the incidence of cancer and increases the survival of cancer patients as well as the response to immunotherapies. In the murine B16 melanoma model, exercise has been shown to significantly reduce the tumor incidence, which was accompanied by an increased infiltration of immune cells into tumors, in particular NK cells and CD8^+^ cytotoxic T lymphocytes (CTL). This can be associated with an increase of (nor)epinephrine serum levels, leading to the mobilization of immune cells. A randomized controlled clinical trial comprising of 56 lung cancer patients was designed with patients in the exercise arm receiving three high intensity exercise sessions/week for six weeks (NCT04263467, PMID: 35,247,994). This results in an immune cell mobilization of these patients, a reduced toxicity of chemo- and immunotherapy as well as reduced hospital admissions. The exercise-mediated increase in catecholamines was associated with higher numbers of NK cells as well as circulating T cells and B cells, which was accompanied by an unrelated patients’ survival. Acute high levels of adrenaline/noradrenaline induced by exercise reduces cancer risk, increases survival and enhances the response to ICPi, while stress- or tumor-induced noradrenaline contributes to an immune suppressive TME. Analysis of the physiological adrenaline concentrations on the function of T and NK cells demonstrated a negative impact of chronic adrenergic signaling on the IFN-γ secretion of PBMCs.

A novel pMHC-targeting approach was provided by Kristoffer H. Johansen from the Technical University of Denmark in Lyngby, Denmark. He presented a rapid artificial intelligence (AI)-generated de-novo pMHC minibinder design platform, which allowed targeting of cancer-associated peptides presented by the MHC complex. Using a computational pre-screening of specificity and molecular dynamics simulation allowed a better predictability of minibinders binding to specific target antigens. He further demonstrated that such targeting could be done to neoantigen pMHCs for which no solved structure exists. This is an important approach since the identification of TCR from patient material is complex. A high affinity tumor antigen minibinder was identified and its structure was confirmed by cryo-electron microscopy. Furthermore, this binder induced killing of tumor cells and showed some peptide selectivity. This novel approach facilitates a rapid design and validation of proteins that could recognize peptides from, e.g., tumor-associated proteins or neoantigens with a high specificity and low off-target recognition.

Another interesting approach is the use of the “dark matter” of cancer presented by Bernard Fox from the Earle A. Chiles Research Institute in Portland, USA. These non-canonical peptides could be presented by HLA-I molecules, but a lack of reactivity to these antigens exists by endogenous tumor-reactive T cells. However, T cells derived from both peripheral blood or TILs and generated in vitro by stimulation with non-canonical peptides were able to recognize HLA-presented epitopes on the majority of cancer cells, thereby providing novel opportunities for the treatment of tumor patients. These non-canonical peptides could be derived from open reading frames, the 5’ UTR as well as endogenous retroelements. Since most of the non-canonical peptides are short-lived proteins or defective ribosomal products, they are not subject to thymic selection and typically degraded within 30 min, but efficiently captured by HLA-I molecules and widely expressed on the tumor cell surface. So far, only neoantigens from long-lived proteins of tumor cells have been used as vaccines, while the non-canonic epitopes are not cross-presented due to their short-lived nature and failed to induce therapeutic responses.

Next to non-canonical peptides as targets, the combination of tyrosine kinase inhibitors (TKIs) with different immunotherapies as well as CAR T cell therapy is a promising. Christine Dierks from the University Hospital of the Martin-Luther-University in Halle, Germany, talked about thyroid carcinomas (TC) and the clinical need for novel treatment options of TC subsets due to their worse ten years’ prognosis. Interestingly, the TMB has been associated in TCs with an increased PD-L1 expression. Anaplastic thyroid carcinoma (ATC), the most aggressive TC tumor type with a five months median survival, is characterized by a high proliferation rate, increased neoangiogenesis, a high frequency of mutations and high levels of PD-L1 expression. A combination of angiogenesis inhibitors with ICPis demonstrated a good response rate of ATC patients and poorly differentiated thyroid cancer (PDTC). The tyrosine protein kinase transmembrane receptor (ROR)1 is overexpressed in ATC and PDTC as well as in other solid tumor entities, but not expressed in most normal tissues. Since ROR1 overexpression in tumors were associated with the patients’ survival, ROR1 represents a suitable target. Therefore, a ROR1-CAR construct was developed and tested in vitro using 2D and 3D models and in vivo. ROR1-CAR T cells were highly specific and able to lyse small tumors in vivo. However, CAR T cell therapy often fails due to poor tumor infiltration and an immune suppressive TME, T cell exhaustion and downregulation of target, while TKIs known to block the angiogenesis can also promote T cell infiltration and inhibit tumor growth. Treatment of TKIs in combination with CAR T cells demonstrated an improved efficacy in vitro as well as a reduced tumor growth in vivo, which was further improved by multiple CAR T cell injections. ROR1-CAR T cells, but not TKIs reduced metastasis development in vivo as well as the number of circulating tumor cells, while TKI alone increased the number of circulating ROR1-CAR T cells in vivo and changed the CAR T cell profile. The combination enhanced T cell activation, reduced exhaustion of circulating CAR T cells and increased the number of effector and effector memory T cells in vivo. In addition, TKIs influenced the cellular TME composition by decreasing the immune suppressive CAF-S1 subtype. In sum, the combination of TKI with CAR T cells reduced tumor growth, eliminate circulating tumor cells thereby blocking metastasis formation, while TKI treatment alone resulted in an upregulation of immune response pathways and circulating CAR T cells accompanied by an inhibition of tumor growth as well as reversion of the immune suppressive TME.

The use of CAR T cells, BiTEs and other emerging strategies unleashing T cells against AML was reported by Marion Subklewe from the LMU in Munich, Germany. Since allogenic stem cell transplantation (SCT) is the most effective treatment in AML, the success of allo-SCT was translated to T cell-based synthetic immunity in AML. However, one major challenge in AML is the identification of suitable target antigens and the mechanisms of resistance suggesting that combinatorial strategies might be the future for the treatment of patients with low AML burden. The target antigens could be cell type specific, lineage-restricted, leukemia-associated or leukemia-specific and ideally expressed in most AML cells, critical for AML biology, but absent on vital healthy cells. So far, the lineage-restricted myeloid antigens CD33, CD123 and CLL-1 are the most commonly targeted antigens in this disease. However, it is noteworthy that (i) continuous antigen exposure could lead to T cell exhaustion, (ii) the antigen expression is very heterogeneous in bulk AML cells and (iii) escape variants could develop with high inter- and intra-individual expression profiles. Therefore, suitable target antigens in AML are required and were identified by analyses of the AML surfaceome, which could be targeted by Abs. So far, a number of BiTEs targeting CD3ε together with CD19, CD20, BCMA or GPRC, respectively, have been developed and demonstrated a high efficacy and durable response in B cell and plasma malignancies. Since these BiTEs had a low efficacy and no sustained responses in AML, more restricted target antigens in particular intracellular antigens are required. These include bispecific TCR mimicry molecules targeting, e.g., GP100, PRAME, MAGE, survivin Wilms tumor 1 and common TP53 mutations. Resistances to T cell-recruiting bispecific Abs including tumor intrinsic mechanisms, but also extrinsic strategies like T cell dysfunction as well as the immune suppressive TME exist. T cells at relapse exhibit a memory and exhaustion associated transcriptional and epigenetic profiles compared to T cells at initial diagnosis, characterized by a senescence profile. Since T cells lack a metabolic fitness upon continuous stimulation with a CD33 TCR, T cell exhaustion is induced by continuous exposure suggesting treatment-free intervals ameliorate T cell exhaustion. Current approaches to maintain T cell function include ICP blockade, but also genetic engineering, epigenetic modulation, metabolic modulation as well as cytokine supplementation.

Michael Bachmann from the HZDR in Dresden, Germany, summarized the current data set available for the switchable adaptor CAR T cell platforms known as UniCAR and RevCAR system. In contrast to permanently active conventional CAR T cells, adaptor CAR T cells are only functional in the presence of a bispecific targeting module (TM) which mediates a cross-linkage between the universal CAR T cells with the respective target cell. Over the past decades, a series of either antibody-derived or small molecule (e.g., tracer)-based TMs for targeting of tumor-associated antigens expressed on either leukemic cells (e.g., CD33, CD123) or solid tumors (e.g., prostate stem cell antigen, prostate membrane antigen) were established for which in part proof of functionality was tested and confirmed in clinical phase 1 trials. In addition, TMs for targeting components of the TME including the fibroblast activating protein expressed on CAFs were developed. The different TMs can be applied either separately or in parallel and thereby enable a multispecific including gated targeting of tumor tissues. In 3D cell and animal models, the simultaneous targeting of tumor antigens and the TME was found to enhance the entrance of CAR T cells into solid tumors and thus, the efficacy of CAR T cells. Further improvements can be achieved by radiolabelling of TMs with either diagnostic (e.g., ^64^Cu, ^177^Lut), therapeutic (e.g., ^177^Lut, actinium) or theragnostic radionuclides (e.g., ^177^Lut). In dependence on the respective radionuclide, the radiolabelled TMs can be used for imaging of tumor tissues to follow CAR T cell therapies with positron emission tomography or single-photon emission computed tomography or applied for a combination of endoradionuclide therapy with (CAR T cell-based) immunotherapies. First experimental evidence supports the hypothesis that such a combinatorial radio-/immunotheranostic approach helps to overcome the immunosuppressive TME of solid tumors.

Stina Wickström from the Karolinska Institute in Stockholm in Sweden presented a study of melanoma patients with TILs using ACT and a vaccine based on DC. In order to improve efficacy of TIL therapy, her team implemented a unique treatment strategy by combining TIL therapy with DC-based cancer vaccines. The first patients demonstrated encouraging results with strong partial or complete responses. Since the TME as well as reactive oxygen species (ROS) play a role in tumor progression, a strategy to render human CD8^+^ CTLs resistant toward ROS was implemented by activation of Nrf2 accompanied by intrinsic anti-oxidant responses. Based on first preliminary results, S. Wickström hypothesized that Nrf2 activation in TILs prior to ACT might enhance the treatment efficacy of these patients. Furthermore, the DC vaccine using whole tumor lysates was improved by modification of the pre-treatment protocols of tumor cells prior to the generation of the tumor lysate, which enhances the capacity of DC vaccines to activate CD8^+^ T cells. Current studies are on the way to improve the DC vaccine efficacy by its combination with different ICPis.

Douglas McNeel from the University Wisconsin in Madison, USA, talked about a clinical trial for the treatment of patients with high-risk PCa, which is based on androgen deprivation leading to increased androgen receptor (AR) expression in PCa cells and thus a better recognition of AR-specific CD8^+^ T cells. Since DNA vaccination encoding AR LBD led to an increased tumor immune cell infiltration as well as activation of PD1-expressing CD8^+^ T cells, he suggested the combination of androgen deprivation and AR-targeted vaccination and ICPi treatment to increase tumor-specific CD8^+^ T cell infiltration, tumor eradication as well as immune memory. Only few adverse effects were found upon AR-targeted vaccination in the absence of PD-1 blockade with a modest peripheral T cell response to the vaccine antigen, which was still detectable six months after vaccination following prostatectomy also leading to an increased CD8^+^ T cell infiltration in PCa lesions. Androgen deprivation which was enhanced upon combination with vaccination increased B cells and activated CD8^+^ T cells and decreased CD4^+^ CTLA4^+^ T cells. In the next step, timing of the PD-1 blockade with vaccine was monitored with a PD-1 blockade at the time of initial priming and vaccination. Simultaneously delivery of LAG3 and PD-1 blockade suggested a further benefit. In the HLA-A2/TRAMP PCa model, vaccination was combined with anti-PD-1 treatment and AR depletion. IHC analyses of tissues as well as spleens demonstrated an increased T cell infiltration and alterations in B cells, MDSCs as well as Tregs upon androgen deprivation. Following androgen deprivation the TILs have a distinct memory effector phenotype, an altered expression of ICP as well as changes in the immune suppressive activity. Based on these results, the treatment arm with the highest efficacy will be expanded and further patients will be recruited followed by evaluation of the antigen specificity as well as the transcriptional profile of PCa tissue infiltrating lymphocytes. In addition to this, PCa tissue infiltrating CD8^+^ T cells are currently being expanded ex vivo, to evaluate their function and whether these might be used in long-term for ACT of patients with recurrent disease.

In conclusion, the TIMO 2025 conference demonstrated that tumor immunology is a dynamic and rapidly growing field that continues to gain significance. The discussions and findings from the conference are poised to serve as a catalyst for future research and clinical applications aimed at improving outcomes for cancer patients worldwide. Future developments in tumor immunology will be significantly influenced by the topics discussed during the TIMO conference. The following issues provide important perspectives and have to be developed: These include (i) in-depth research on immune escape mechanisms, which might lead to the design of novel therapeutic strategies, (ii) integration of “omics” technologies, which facilitate the identification of therapeutic targets, (iii) development of personalized immunotherapies through the identification of specific biomarkers, (iv) interdisciplinary approaches, which will be vital for obtaining a comprehensive understanding of tumor biology and establishing innovative therapies and (v) optimization of combination therapies to enhance treatment efficacy and improve patients’ survival rates.

## Ethical approval

Not applicable.

## Data Availability

No datasets were generated or analysed during the current study.

## Abbreviations

Ab: Antibody
ACT: Adoptive cell therapy
AI: Artificial intelligence
ALL: Acute lymphatic leukemia
AML: Acute myeloid leukemia
AR: Androgen receptor
ATC: Anaplastic thyroid carcinoma
BiTE: T cell engaging bispecific antibody
CAF: Cancer-associated fibroblast
CAR: Chimeric antigen receptor
CIITA: Class II major histocompatibility complex transactivator
CRC: Colorectal cancer
CTL: Cytotoxic T lymphocytes
CTLA-4: Cytotoxic T lymphocyte associated protein 4
FLT3: Fms-like tyrosine kinase 3
GVHD: Graft versus host disease
GvL: Graft versus leukemia
ECM: Extracellular matrix
HIF: Hypoxia-inducible factor
HLA: Human leukocyte antigen
HSCT: Hematopoietic stem cell transplantation
ICP: Immune checkpoint
ICPi: Immune checkpoint inhibitor
IDO: Indolamine 2, 3-deoxygenase
IFN: Interferon
IHC: Immunohistochemistry
IL-2R: IL-2 receptor
Kyn: Kynurenine
LRA: Lineage-restricted antigens
mAb: Monoclonal antibody
miBd: Minibinder
miRNA: MicroRNA
MOMP: Mitochondrial outer membrane permeabilization
MSI: Microsatellite instability
NK: Natural killer
OC: Ovarian cancer
PARP: Polyadenosine-diphosphate-ribose polymerase
PBMC: Peripheral mononuclear cell
PCa: Prostate cancer
PD-1: Programmed death receptor 1
PDAC: Pancreatic adenocarcinoma
PD-L1: Programmed death receptor ligand 1
PDO: Patient-derived organoid
PDTC: Poorly differentiated thyroid cancer
PMN-MDSC: Polymorphonuclear myeloid-derived suppressor cells
PSMA: Prostate specific membrane antigen
RNAseq: RNA sequencing
ROR: Tyrosine protein kinase transmembrane receptor
ROS: Reactive oxygen species
scRNA-seq: Single cell RNAseq
SCT: Stem cell transplantation
TAM: Tumor-associated macrophages
TC: Thyroid carcinoma
TCR: T cell receptor
TGF: Transforming growth factor
TIGIT: T cell immunoglobulin and immunoreceptor tyrosine-based inhibitory motif
TIL: Tumor infiltrating lymphocyte
TIM-3: T cell immunoglobulin and mucin domain-3
TIMO: Tumor immunology meets oncology
TKI: Tyrosine kinase inhibitor
TM: Targeting molecule
TMB: Tumor mutational burden
TME: Tumor microenvironment
TNBC: Triple negative breast cancer
Treg: Regulatory T cell

